# Strasseriolides display *in vitro* and *in vivo* activity against trypanosomal parasites and cause morphological and size defects in *Trypanosoma cruzi*

**DOI:** 10.1371/journal.pntd.0011592

**Published:** 2023-09-15

**Authors:** Cristina Bosch-Navarrete, Guiomar Pérez-Moreno, Frederick Annang, Rosario Diaz-Gonzalez, Raquel García-Hernández, Hedy Rocha, Francisco Gamarro, Carlos Cordón-Obras, Miguel Navarro, Ana Rodriguez, Olga Genilloud, Fernando Reyes, Francisca Vicente, Luis M. Ruiz-Pérez, Dolores González-Pacanowska

**Affiliations:** 1 Instituto de Parasitología y Biomedicina "López-Neyra", Consejo Superior de Investigaciones Científicas (CSIC), Parque Tecnológico Ciencias de la Salud, Granada, Spain; 2 Fundación MEDINA, Parque Tecnológico Ciencias de la Salud, Granada, Spain; 3 Department of Microbiology, Core Anti-infectives, New York University School of Medicine, New York, New York, United States of America; Hebrew University-Hadassah Medical School, ISRAEL

## Abstract

Neglected diseases caused by kinetoplastid parasites are a health burden in tropical and subtropical countries. The need to create safe and effective medicines to improve treatment remains a priority. Microbial natural products are a source of chemical diversity that provides a valuable approach for identifying new drug candidates. We recently reported the discovery and bioassay-guided isolation of a novel family of macrolides with antiplasmodial activity. The novel family of four potent antimalarial macrolides, strasseriolides A-D, was isolated from cultures of *Strasseria geniculata* CF-247251, a fungal strain obtained from plant tissues. In the present study, we analyze these strasseriolides for activity against kinetoplastid protozoan parasites, namely, *Trypanosoma brucei brucei*, *Leishmania donovani* and *Trypanosoma cruzi*. Compounds exhibited mostly low activities against *T*. *b*. *brucei*, yet notable growth inhibition and selectivity were observed for strasseriolides C and D in the clinically relevant intracellular *T*. *cruzi* and *L*. *donovani* amastigotes with EC_50_ values in the low micromolar range. Compound C is fast-acting and active against both intracellular and trypomastigote forms of *T*. *cruzi*. While cell cycle defects were not identified, prominent morphological changes were visualized by differential interference contrast microscopy and smaller and rounded parasites were visualized upon exposure to strasseriolide C. Moreover, compound C lowers parasitaemia *in vivo* in acute models of infection of Chagas disease. Hence, strasseriolide C is a novel natural product active against different forms of *T*. *cruzi in vitro* and *in vivo*. The study provides an avenue for blocking infection of new cells, a strategy that could additionally contribute to avoid treatment failure.

## Author summary

Kinetoplastid parasites are a health burden in tropical and subtropical countries. Important human diseases produced by kinetoplastids include Human African trypanosomiasis which is caused by the protozoan *Trypanosoma brucei*, Chagas disease, due to infection with *Trypanosoma cruzi* and the leishmaniases which are caused by infection with parasites of the *Leishmania* genus. We previously reported the discovery and isolation of a novel family of four macrolides, produced by the fungus *Strasseria geniculata* with antiplasmodial activity. In the present study we analyze these strasseriolides for activity against kinetoplastid parasites. Out of the four strasseriolides tested, C and D were active against intracellular *T*. *cruzi* and *L*. *donovani* amastigotes with EC_50_ values in the low micromolar range. Compound C is fast-acting against both intracellular amastigotes and extracellular trypomastigote forms of *T*. *cruzi* cultured *in vitro* by infecting mammalian cells, and lowers parasitaemia in acute models of infection of Chagas disease. Hence, strasseriolide C is a promising candidate for the development of new therapeutic strategies to treat kinetoplastid diseases.

## Introduction

Leishmaniasis, trypanosomiasis, and Chagas disease are infectious tropical vector-borne diseases caused by kinetoplastid protozoan parasites. Their devastating impact on vulnerable populations in developing countries together with an insufficient investment in research programs, situate them among the group termed *Neglected Tropical Diseases* (NTDs). Progress against NTDs has alleviated the human and economic burden but 1.74 billion people worldwide still require future interventions.

Leishmaniasis is caused by 20 different species of protozoan parasites of the genus *Leishmania* which are transmitted by the bite of infected phlebotomine sandflies. There are three main disease forms: visceral (also known as kala-azar), cutaneous and mucocutaneous leishmaniasis. An estimated 700,000 to 1 million new cases of leishmaniasis occur annually [1]. Despite their severe side effects, antimonials have been used to treat the disease for nearly 70 years. Shorter, affordable, safer, and more easily administered alternative treatments are required to replace amphotericin B and miltefosine.

American Trypanosomiasis, also known as Chagas disease, is a systemic disease caused by the protozoan *Trypanosoma cruzi* transmitted by triatomine arthropods. Chagas disease is the most prevalent communicable tropical disease in Latin America. It is endemic in 21 countries in the Americas and affects approximately 6 million people, causing 12,000 deaths per year [2]. For Chagas disease there is a lack of adequate chemotherapy; nifurtimox and benznidazole dosages eventually result in adverse effects and reduced efficacy after long treatments. Few compounds have entered clinical trials and repositioned antifungal azoles have unfortunately limitations. Quiescent forms of *T*. *cruzi* further complicate a complete cure [3]. At present, most efforts are centered in the identification of shorter treatment regimens that are at least as effective as today’s standard treatment with benznidazole, but with fewer side effects. After posaconazole failure in clinical trials [4], fexinidazole, the latest approved drug treatment for sleeping sickness, is being evaluated in a phase II proof-of-concept study as a potential drug for Chagas disease [5] while several ongoing hit-to-lead collaborative programmes pursue the discovery of new active molecules excluding those that inhibit the CYP51 enzyme [6–9].

Human African trypanosomiasis (HAT), commonly called sleeping sickness, is caused by two subspecies of the genus parasite *Trypanosoma brucei*, *T*. *b*. *gambiense* and *T*. *b*. *rhodesiense*, which are transmitted by tsetse flies. HAT is endemic in sub-Saharan countries and annual number of cases has fallen from more than 7,000 in 2012 to fewer than 1,000 in 2019 [10] with more than 98% of infections attributed to *T*. *b*. *gambiense*. For decades, the only treatments available for second-stage HAT were melarsoprol and more recently eflornithine, which is safer though it requires long treatments and parenteral administration. Eflornithine can be used in monotherapy but currently, combined with nifurtimox (NECT) reduces perfusions’ frequency and stands as the first-line treatment for the advanced stage of this disease. Fexinidazole was approved in 2021 as the first oral treatment for both stages of *T*. *b*. *gambiense* sleeping sickness. At present, acoziborole, a single-dose oral treatment, is being evaluated in clinical trials [11].

Natural products represent a rich source of diverse, unexplored and affordable chemical starting points for drug discovery. Many antiparasitic and specifically, antiprotozoal drugs are natural products such as the traditional antimalarials quinine, chloroquine and artemisinin. More recently, the isolation of compounds from marine organisms and plants has yielded novel trypanocidal and antileishmanial molecules such as quinones, sesquiterpenes lactones, flavonoids, peroxides and various alkaloids with no or low cytotoxicity. Different classes of natural products derived from microbial sources have shown potential as antitrypanosomal and antileishmanial agents [12–25].

Previously, a robust HTS platform was implemented for the identification of novel active molecules, isolated from microbial extracts, against HAT, leishmaniasis, Chagas disease [26], and malaria [27]. As a result of this effort, we recently reported the discovery and bioassay-guided isolation of a novel family of four macrolides, produced by the fungus *Strasseria geniculata* with antimalarial activity [28]. These compounds are potent inhibitors of intraerythrocytic stages of *Plasmodium falciparum* and two of them showed a promising pharmacokinetic profile and *in vivo* efficacy in a murine model of the disease [29].

Here we report the promising activity of these macrolides against kinetoplastid protozoan parasites, namely, *T*. *b*. *brucei*, *L*. *donovani* and *T*. *cruzi* and analyze the occurrence upon exposure of potential perturbations in cell cycle and parasite morphology. This work also highlights differences in the mode of action profile between these newly discovered molecules and a set of reference drugs active against kinetoplastids.

## Materials and methods

### Ethics statement

*In vivo* efficacy was monitored at New York University School of Medicine (US). This study was carried out in accordance with the recommendations in the Guide for the Care and Use of Laboratory Animals of the National Institutes of Health (US). The protocol was approved by the Institutional Animal Care and Use Committee of New York University School of Medicine, which is fully accredited by the Association for Assessment and Accreditation of Laboratory Animal Care International.

### Reagents

Fetal bovine serum (FBS) and RPMI 1640 media, sodium pyruvate, penicillin-streptomycin and L-glutamine, were purchased from Invitrogen Gibco, Incl. LIT, RPMI 1640-modified medium and HMI-9 media were prepared at Tissue culture facility in Instituto de Parasitología y Biomedicina “López-Neyra”. Surfact-Amps NP40 was supplied by Thermo Scientific and Tween 20 by Merck. Sodium dodecyl sulfate (SDS) was purchased from Affymetrix. Chlorophenol red-β-D-galactopyranoside (CPRG), resazurin sodium salt, amphotericin B, *p*-formaldehyde, pentamidine isethionate salt, benznidazole, nifurtimox, posaconazole, ATP disodium salt, propidium iodide, podophyllotoxin, ribonuclease A (RNase A) from bovine pancreas, phorbol 12-myristate 13-acetate (PMA),3-(4,5-dimethylthiazol-2-yl)-2,5-diphenyltetrazolium bromide (MTT) and dimethyl sulfoxide (DMSO) were obtained from Sigma Aldrich. CellTiter-Glo Luminescent Cell Viability Assay was purchased from Promega Corporation and 4′, 6-diamidino-2-phenylindole (DAPI) Prolong from Molecular Probes.

### Strains and media

Parasites strains adapted to culture *in vitro* were used. *T*. *b*. *brucei* Lister 427 bloodstream form parasites (information about the identity and genealogy of this strain is available at The Rockefeller University website [30]) were grown in HMI-9 medium supplemented with 10% heat-inactivated fetal bovine serum (iFBS).

The *Trypanosoma cruzi* Tulahuen strain used is a genetically modified strain that expresses the *Escherichia coli* β-galactosidase gene, lacZ [31]. It was kindly supplied by Marcel Kaiser (Swiss Tropical and Public Health Institute). The *T*. *cruzi* Tulahuen C4 strain was cultured in RPMI-1640 supplemented with 10% inactivated FBS (iFBS), 1.7 mM L-glutamine, 100 U/mL penicillin, and 100 U/mL streptomycin at 37°C and 5% CO_2_. L6 rat skeletal muscle cells were purchased from ATCC (reference CRL-1458) and used as host cells for infection with transgenic *T*. *cruzi* trypomastigotes. Epimastigotes were obtained by differentiation in LIT medium with 10% iFBS at 28°C from trypomastigote forms.

*Leishmania donovani* MHOM/ET/67/HU3 cells with the luciferase gene integrated into the parasite genome was obtained as described [32]. Parasites were grown at 28°C in RPMI 1640-modified medium supplemented with 20% heat-inactivated fetal bovine serum (iFBS, Invitrogen) with 100 μmg/mL of hygromycin B.

The human myelomonocytic cell line THP-1 (from ATCC, reference TIB-202) was grown at 37°C and 5% CO_2_ in RPMI-1640 supplemented with 10% iFBS, 2 mM glutamate, 100 μ/mL penicillin and 100 mg/mL streptomycin.

### *T*. *cruzi* trypomastigote viability assay

Tulahuen *T*. *cruzi* trypomastigotes were recovered 6 days post infection from the supernatant of harvested infected L6 cells cultures after 3 hours at 37°C. 90 μL of parasite culture at a density of 5 × 10^6^ cells in RPMI 1640 media were dispensed into 96 Nunc flat bottom assay plates containing 10 μL of each compound.

Parasite viability was assessed upon 24, 48 and 72 hour incubation at 37°C in 5% CO_2_ by adding 50 μL of CellTiter-Glo viability reagent to white 96-well microplates. After signal stabilization, bioluminescence was read. Compound concentrations ranged from 0.1 μM to 50 μM and final maximal DMSO percentage in the well was 0.05%. Results were normalized to negative (50 μM nifurtimox) and positive control columns in each plate.

In the fluorescence assay, plates were incubated for 24 and 48 hours after which 10 μL of the resazurin solution were added to each well. Plates were further incubated for 24 hours and fluorescence was measured at 550/590 nm using a Tecan Infinite 200 microplate reader. Benznidazole at 400 μM was used as 100% loss of viability control and analyzed at decreasing concentrations to confirm the reported EC_50_ value [7,33]. Final maximal DMSO percentage in the well was 0.1% and compound concentrations ranged from 0.2 to 100 μM.

### *T*. *cruzi* epimastigotes growth inhibition assay

A resazurin-based assay was set up in 96-well microplates containing 10 μL of test compound at concentrations ranging from 0.39 to 200 μM and log phase parasites seeded at 2 × 10^5^ cells per well in a final volume of 100 μL. Maximum DMSO percentage in the well was 0.2%. Positive and negative growth controls were included. Following 72 hour incubation at 28°C with orbital shaking, 10 μL of resazurin solution were added to each well and the plates were returned to the incubator for 4 hours. Resazurin reduction was correlated to cell viability by fluorescence measurement with excitation and emission wavelength bands centered at 550 and 590 nm, respectively, in a Tecan Infinite 200 microplate reader. Benznidazole at 400 μM was used as negative growth control and at decreasing concentrations to confirm the reported EC_50_ value [33,34].

### Quantification of ATP intracellular levels

ATP was determined using the CellTiter-Glo assay. Optimum readout conditions and linearity of the assay were established for *T*. *cruzi* epimastigotes by testing different cell densities. The assay was linear up to 100,000 cells (in 90 μl) per well. 50,000 cells (from log phase cultures) per well were dispensed into 96-well white flat bottom microplates containing 10 μl of strasseriolide C at concentrations ranging from 0.4 to 6.25 μM and grown at 28°C for 72 hours with orbital shaking. ATP standard curves were obtained using concentrations ranging from 0.1 to 100 pmol of ATP disodium salt in 90 μl of LIT medium per well. Both samples and standards were assayed in triplicate. 70 μL of CellTiter-Glo solution were added to treated and control parasites and to the standard curve wells, and luminescence detection was recorded after 15 minutes incubation in a Tecan Infinite 200 microplate reader.

### β-D-galactosidase transgenic *T*. *cruzi* assay

A Thermo Scientific Multidrop Combi dispenser was used to dispense 55 μL of *T*. *cruzi* infected L6 cells (2 ×10^3^ cells per well) into 384-well Corning assay plates already containing 5 μL of each compound at concentrations ranging from 0.0015 to 50 μM and controls. The plates were incubated at 37°C for 96 hours upon which 15 μL of lysis buffer (0.5 mM CPRG, 0.5% NP40) were added to each well and further incubated for 4 hours in the dark. Absorbance at 585 nm was measured in a Perkin Elmer Envision plate reader. Benznidazole at 40 μM was used as negative growth control.

### Rate of kill

For rate of kill experiments, *in vitro* activity against intracellular *T*. *cruzi* amastigotes was analyzed at early time points: 24, 48 and 72 hours. The standard colorimetric transgenic assay was established in a 96-well format, with serial dilutions of compounds and 0.05% as the final maximal DMSO percentage per well. In these experiments L6 host cells were included as negative controls and CPRG conversion was monitored at 585 nm using a Versamax microplate reader (Molecular devices).

### Resazurin-based *T*. *b*. *brucei* assay

Cells in logarithmic phase of growth were added using a Multidrop dispenser (45 μL, 500 bloodstream *T*. *b*. *brucei* parasites per well) to 384-well Corning assay plates already containing 5 μL of each compound and controls, and incubated for 72 hours at 37°C. 10 μL of resazurin were added per well, and the plates were further incubated for 6 hours at 37°C. The final fluorescence was determined at 550–590 nm. Pentamidine at 40 nM and 0.0067% DMSO were used as negative and positive growth controls, respectively.

### *L*. *donovani* intracellular amastigote assay

THP-1 cells were differentiated into macrophages with 20 ng/mL of PMA in a 96-well plate at 3 × 10^4^ cells per well for 48 hours followed by 24 hours of culture in fresh medium. Cells were then infected with transgenic stationary *L*. *donovani* MHOM/ET/67/HU3 promastigotes expressing the luciferase gene at a multiplicity of infection (MOI) of 10. A volume of 100 μL of culture media containing the test compounds was added to each well and the plates were incubated at 37°C for 72 hours. Luminescence was measured using the Promega kit luciferase assay system.

### Cytotoxicity evaluation

One hundred microliters of supplemented RPMI 1640 medium containing the compounds and controls were added to L6 rat myoblast cells previously seeded (4,000 cells per well) in a 96-well microplate. After 72 hours at 37°C the viable cell number was determined by resazurin reduction. 20 μL of resazurin at 0.11 mg/mL in PBS 1X were added to each well and incubated in the dark for 2 hours at 37°C. Cell viability was estimated by measuring the final fluorescence at 570–590 nm in a Tecan Infinite F200 plate reader.

Cellular toxicity of compounds in the human macrophage host TPH-1 cell line was determined using the colorimetric MTT-based assay as previously described [35].

THP-1 cells were seeded at 3 × 10^4^ cells/well in 96-well plates and were differentiated to macrophages with 20 ng/ml of PMA treatment for 48 h followed by 24 h incubation in fresh medium. Strasseriolides were added at increasing concentrations up to 50 μM and cells were further incubated at 37°C for 72 h. 10 μL of MTT (5 mg/mL in PBS) were added to each well, and plates were incubated for an additional period of 8 h at 37°C. Formazan crystals were dissolved by adding 50 μL of 20% SDS and absorbance was read at 595 nm using a microplate reader.

### Fluorescence-activated cell sorting (FACS) analysis

FACS analysis was used to study cell cycle progression and cellular parameters such as size and complexity. 5 × 10^6^ *T*. *cruzi* epimastigotes were harvested by centrifugation at 1,000×g and 4°C after 72 hours of incubation in a 6-well plate in the presence and in absence of test compounds. Pellets of cells were washed with 5 mL of PBS and fixed in a solution of 70% ice-cold ethanol/PBS at -20°C overnight. Cells were then collected by centrifugation, washed with PBS and stained with 40 μg/mL of propidium iodide in PBS containing 10 μg/mL of RNase A at 37°C for 30 minutes. Fluorescence was monitored with a Becton Dickinson FACSCalibur using BD CellQuest Pro version 4.0.2 software with a total of 10,000 events acquired for each sample. The different cell populations as well as mean cellular parameters were determined by FlowJo software.

### Microscopy analysis

For morphological phenotypic analysis and further confirmation of cell cycle analysis by FACS, *T*. *cruzi* epimastigotes were grown for 72 hours in a 6-well plate in the presence and absence of drugs. Briefly, 1 × 10^6^ parasites of each condition were harvested and fixed in 4% *p*-formaldehyde at 4°C for 20 minutes, washed twice with cold PBS, and placed on poly-L-lysine-coated slides. Followed by dehydration in methanol, parasite samples were stained and mounted with DAPI Prolong. Digital images were acquired with a Leica DMi8 microscope in Tile Scan mode with 305 LED excitation to quantify DAPI stained nuclei and kinetoplasts of the parasite population. Transmitted light microscopy was used to acquire differential interference contrast (DIC) images (> 80 individual cells in each of two independent experiments).

### Tested compounds

Samples of the four new macrolides were obtained in a pure form (> 95%, see HPLC traces in S1 Fig) from cultures of the fungal strain, *Strasseria geniculata* CF-247251 as previously described [28]. This fungal strain was isolated as an axenic culture from the inner fragments of the living tissues in the roots of an unnamed plant collected in Mimiha, New Zealand and belongs to Fundación MEDINA’s Collection. Stock solutions of strasseriolides were prepared in DMSO at 100 mM and then further diluted in culture media as necessary. Solutions of nifurtimox, posaconazole and benznidazole were prepared in DMSO at 40, 50 and 192 mM, respectively, and then diluted in culture media to the final working concentration.

### Data analysis

Compound activities were normalized using the in-plate negative and positive controls according to the following equation:

$$Percentage\;inhibition\;growth=\left[1-(\frac{{Abs}_{well}-{Abs}_{neg}}{{Abs}_{pos}-{Abs}_{neg}})\right]\;x\;100$$

where *Abs*_well_ is the absorbance value of a specific well, and *Abs*_pos_ and *Abs*_neg_ are the average absorbances measured for the positive and negative controls, respectively.

The results are expressed as EC_50_ values, meaning the concentration of compound that reduces cell growth by 50% versus untreated control cells.

For 384-well assays, the EC_50_ of each compound was calculated from a 16-point dose response curve while for 96-well assays, 10-point dose-response curves were analyzed with SigmaPlot Software. All EC_50_ values represent the average of at least three biological replicates.

Hill slopes were obtained with SigmaPlot Software from 4-parameter logistic function regression that fits experimental dose-response data while EC_90_ values were calculated with GraphPad Software.

### Statistical analysis

Data are plotted as the mean ±S.D. for each group. IBM SSPS Statistics version 26 was used for comparison of data sets using Student’s t-test. Welch’s two-sample t test was applied when Levene’s normality test or Kolmogorov-Smirnov test failed. A *p* value ≤0.05 is considered statistically significant.

### *In vivo* efficacy experiments

*In vivo* experiments were performed at the Anti-infectives Screening Core Services facility of the New York University School of Medicine. The transgenic *Trypanosoma cruzi* Brazil strain expressing firefly luciferase was used [36,37]. Groups of five female BALB/c mice were infected with 2 × 10^6^ *T*. *cruzi* trypomastigotes. In the treatment group, strasseriolide C treatment started 72 hours post-infection by intraperitoneal (i.p.) dosage of 50 mg/Kg in vehicle (0.5% hydroxymethyl cellulose + 0.5% Tween-80) and was repeatedly continued for five consecutive days. Quantification is based on luciferase activity of the parasites after i.p. injection of 150 mg/Kg D-luciferin potassium-salt in PBS. Luminescence is measured on the fifth day using an IVIS Lumina II imager. Groups treated with vehicle only (0.5% hydroxymethyl cellulose + 0.5% Tween-80) or 30 mg/kg benznidazole i.p. dosage for five days were used as negative and positive controls, respectively.

## Results and discussion

### Antiparasitic activity

Trypanosomatids have complex life cycles, with distinct stages that occur between the insect vector and a vertebrate host. *In vitro* assays against the mammalian stages of *T*. *cruzi*, *L*. *donovani* and *T*. *b*. *brucei* parasites were developed in a 384-well format, using the intracellular amastigote forms of *T*. *cruzi* and *L*. *donovani*, and the bloodstream form of *T*. *b*. *brucei*. The Z-scores obtained for these bioassays were 0.83, 0.70 and 0.74, respectively [26].

**Fig 1:**
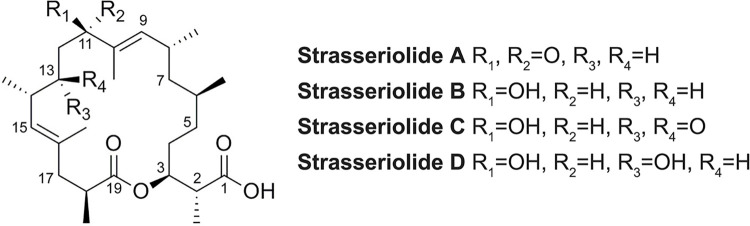
Structures of strasseriolides A-D.

Biological evaluation of the four newly discovered macrolides (Fig 1) in the protozoan parasites *T*. *b*. *brucei*, *L*. *donovani* and *T*. *cruzi* revealed low activity for strasseriolide A while the potency of the highly active strasseriolide B was more than two orders of magnitude higher than that of C or D against both intracellular *L*. *donovani* and *T*. *cruzi* as indicated in Table 1. However B appears as a promiscuous compound and has been reported to be toxic *in vivo* [29].

**Table 1:** EC_50_ values for strasseriolides A-D in protozoan parasites.

Compound	*T*. *brucei* ^a^	*L*. *donovani* ^b^	*T*. *cruzi* ^c^		
			Epimastigotes	Trypomastigotes	Amastigotes
A	273.2	> 15	> 50	> 25	31.0
B	27	0.032	< 0.39	< 0.20	0.21
C	59.6	6.63	32.4	3.84	3.08
D	> 100	13.6	42.4	3.38	5.61
Pentamidine	0.002	─	─	─	─
Amphotericin B	─	0.053	─	─	─
Benznidazole	─	─	13.0	3.93	1.0
Chloroquine	─	─	─	─	─

Data shown are mean values from at least three independent replicates. Reference drugs were used as negative growth control.

(a) *T*. *b*. *brucei 427* bloodstream forms. (b) *L*. *donovani* MHOM/ET/67/HU3 transgenic intracellular amastigotes stably expressing luciferase. (c) *T*. *cruzi* Tulahuen C4 transgenic strain stably expressing beta galactosidase.

**Fig 2:**
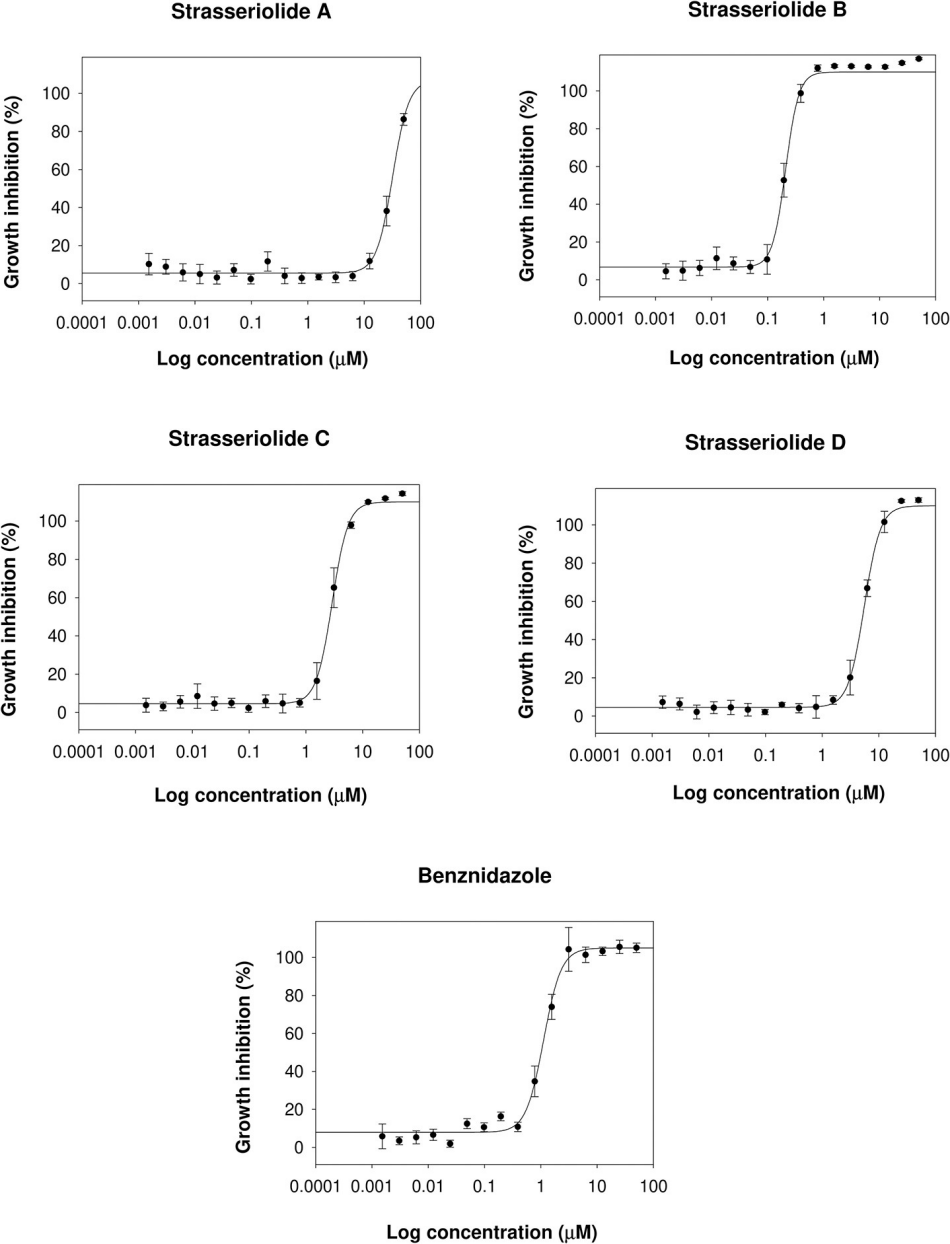
**Dose-response curves for *T*. *cruzi* intracellular amastigotes after 96-hour treatment with strasseriolides A-D.** Growth inhibition has been quantified by CPRG colorimetric read out following the β-D-galactosidase transgenic *T*. *cruzi* assay. Bars represent the mean (± SD) at each of the 16 concentration points calculated from a minimum of three biological replicates.

We have previously shown that four macrolides, strasseriolides A–D, isolated from the fungus *Strasseria geniculata* CF-247251, exhibit EC_50_ values between 0.013 and 9.810 μM against Plasmodium falciparum strain 3D7 whole parasites [28]. In the present study, compounds exhibited mostly low activities against *T*. *b*. *brucei*, yet notable growth inhibition was observed for strasseriolides C and D in the clinically relevant intracellular *T*. *cruzi* (Fig 2) and *L*. *donovani* amastigotes with EC_50_ values for *T*. *cruzi* in the low micromolar range as detailed in Table 1, and hence similar potency to benznidazole [7]. Both HTS assays utilize genetically modified parasites that allow colorimetric and luminescence quantification of proliferation in tissue culture cells [31,38].

Selectivity indexes of compounds C and D calculated as a ratio of inhibitory concentrations versus L6 cells were found to be more than 15-fold for *T*. *cruzi* while in *L*. *donovani* parasites versus TPH-1 host cells, values for strasseriolides C and D were 68 and 37, respectively. These data together with the previously reported assessment of cytotoxicity in HepG2 cell lines [28], underline the overall selective antiprotozoal action of these molecules as summarized in Table 2.

**Table 2:** Cytotoxicity of the four isolated macrolides.

	EC_50_ (μM)	
Compound	L6 (SI)^a^	THP-1 (SI)^b^
A	> 50 (>1)	> 125 (>8)
B	0.30 (1)	161.3 (4962)
C	> 50 (>17)	448 (68)
D	> 100 (>18)	> 500 (>37)

Selectivity indices (SI) are shown in brackets (a) *T*. *cruzi* Tulahuen C4 versus L6 cells. (b) Intracellular *L*. *donovani* MHOM/ET/67/HU3 versus human macrophage THP-1 cells.

Further focus was placed on the extracellular forms of *T*. *cruzi* parasites and trypomastigote and epimastigote assays were set up in a 96-well format after the establishment of optimal conditions for linearity, solvent effect and incubation time (see S2 and S3 Figs and details in Material and Methods) [39].

Thus, the global high activity of B was confirmed in extracellular forms of *T*. *cruzi* with EC_50_ values below 0.4 μM for epimastigotes and lower than 0.2 μM for non-replicative infective trypomastigotes while half maximal effective loss of viability of *T*. *cruzi* trypomastigote cells with strasseriolide A could not be achieved at 25 μM. *In vitro* evaluation of compound A against epimastigote forms of the parasite also indicated moderate efficacy (less than 50% cell growth reduction after 72-hour exposure at a concentration of 50 μM) (Table 1).

Anti-trypomastigote assays are important considering the resistance of this life-stage to different compounds and the potential relationship of lack of efficacy in later phases of *in vivo* evaluation [7]. Further analysis revealed that 3.4 μM of strasseriolide D and 3.8 μM of strasseriolide C results in 50% loss of viability of infective trypomastigotes after 72 hours of incubation at 37°C. Thus, these two compounds, besides acting on intracellular *T*. *cruzi* amastigotes, can kill emerging trypomastigotes parasites after infected human cells collapse, potentially limiting reinfection of healthy host cells.

Both strasseriolides C and D are slightly less effective against *T*. *cruzi* epimastigotes showing moderate activity in the resazurin-based assay that was confirmed by visual counting using a Neubauer chamber and with a luminescence secondary assay. It is worth mentioning that measurable *in vitro* activity against extracellular epimastigotes has been postulated as a distinct characteristic to discriminate CYP51 mode of action molecules [40].

### Strasseriolides are fast-acting *T*. *cruzi* inhibitors

**Fig 3:**
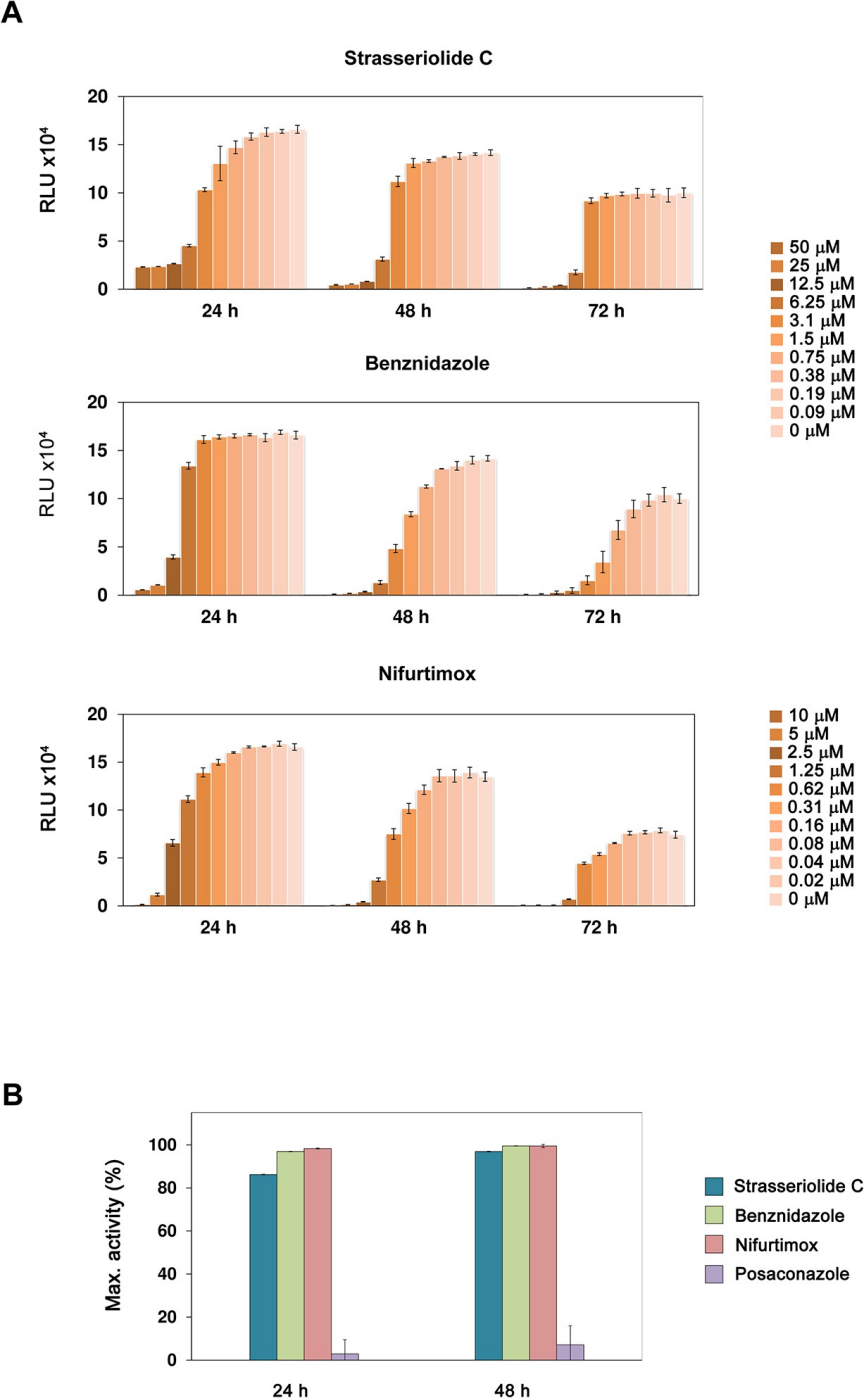
Rate of action against *T*. *cruzi* trypomastigotes. (A) Effect of different concentrations of strasseriolide C, benznidazole and nifurtimox on *T*. *cruzi* trypomastigotes viability. Loss of viability upon 24, 48 and 72 hours drug treatment was measured by the luminescent method at 1/2 serial concentrations ranging from 50 μM strasseriolide and benznidazole and from 10 μM for nifurtimox. Results correspond to the mean value of 3 replicates and error bars represent the standard deviation. (B) Maximum percentage activity against extracellular forms of *T*. *cruzi* reached following 24 and 48 hours treatment with 50 μM strasseriolide C (blue bars), 50 μM benznidazole (green bars), 10 μM nifurtimox (red bars) or 2 μM posaconazole (purple bars) in rate of kill experiments with trypomastigote forms of the parasite.

As shown in Fig 3, the above reported low micromolar trypanocidal activity against non-replicative trypomastigotes was confirmed for compound C even at incubation times as short as 24 hours, by monitoring bioluminescence after the addition of a viability reagent. Posaconazole, nifurtimox and benznidazole were also included as controls in a dose dependent manner for comparison.

The steepness of dose-response curves was quantified by the Hill slope. Strasseriolide C presents a sharp loss of viability profile unlike the reference drugs nifurtimox and benznidazole (Fig 3A) for which activity against *T*. *cruzi* trypomastigotes was gradually dependent on concentration as computed by Hill slopes that were 4.1 and 6.3 at 48 and 72 hours, respectively for compound C, whereas for benznidazole and nifurtimox the slope factor was below 2.1 at both time points (Table 3). The faster behavior of strasseriolide C is translated into a lower EC_90_/EC_50_ ratio: 1.7 and 1.4 for 48 and 72 hours, respectively, versus benznidazole ratios of 3.8 and 3.0 for each of the time points. The nifurtimox EC_90_/EC_50_ ratios were also higher (3.6 and 3.3 at 48 and 72 hours, respectively). The CYP51 inhibitor posaconazole, in contrast, had no effect against these extracellular forms of the parasite (Fig 3B) which is in accordance with previously reported data [7]. The de-prioritization of slow-acting drugs that act on the parasite 14-α-demethylase (TcCYP51), an enzyme involved in the synthesis of sterols, is important considering the failure of azoles such as posaconazole in clinical trials [41]. Although TcCYP51 inhibitors were first identified as promising antichagasic agents, it is now known that their lack of activity on non-replicative trypomastigotes and slow-dividing parasite strains is a reason for this failure [42].

**Table 3:** Drug efficacy of strasseriolide C, benznidazole, nifurtimox and posaconazole against *T*. *cruzi* amastigotes and trypomastigotes.

		Strasseriolide C			Benznidazole			Nifurtimox			Posaconazole		
		EC_50_ (μM)	EC_90_/EC_50_	Hill slope	EC_50_ (μM)	EC_90_/EC_50_	Hill slope	EC_50_ (μM)	EC_90_/EC_50_	Hill slope	EC_50_ (μM)	EC_90_/EC_50_	Hill slope
Amastigotes	**24 h**	10.3	3.78	1.70	2.87	6.28	1.06	0.30	4.07	1.60	5.41	5.16	1.33
	**48 h**	5.25	2.65	2.31	1.03	6.26	1.24	0.15	4.16	1.55	0.0008	6.41	1.20
	**72 h**	5.30	2.43	2.48	0.89	3.07	1.99	0.13	3.03	1.98	0.0007	1.83	3.65
Trypomastigotes	**24 h**	3.29	3.20	2.11	8.94	1.75	3.93	2.08	4.04	1.58	NA	—	—
	**48 h**	4.45	1.72	4.10	2.05	3.75	1.66	0.64	3.61	1.71	NA	—	—
	**72 h**	4.77	1.42	6.31	1.15	2.98	2.07	0.60	3.33	1.83	NA	—	—

Estimated EC_50_ data are mean values from three biological replicates. All standard deviation values are below 20%. Amastigote rate of kill assay uses colorimetric detection (CPRG) while trypomastigote assay read out is bioluminescence. Non-infected L6 cells and nifurtimox are used respectively as negative controls.

Table 3 summarizes strasseriolide C and reference EC_50_ values for trypomastigotes at increasing exposure times which are consistent with those obtained in the resazurin-based assay. Hill slopes and EC_90_/EC_50_ ratios are also indicated for each of the drugs.

**Fig 4:**
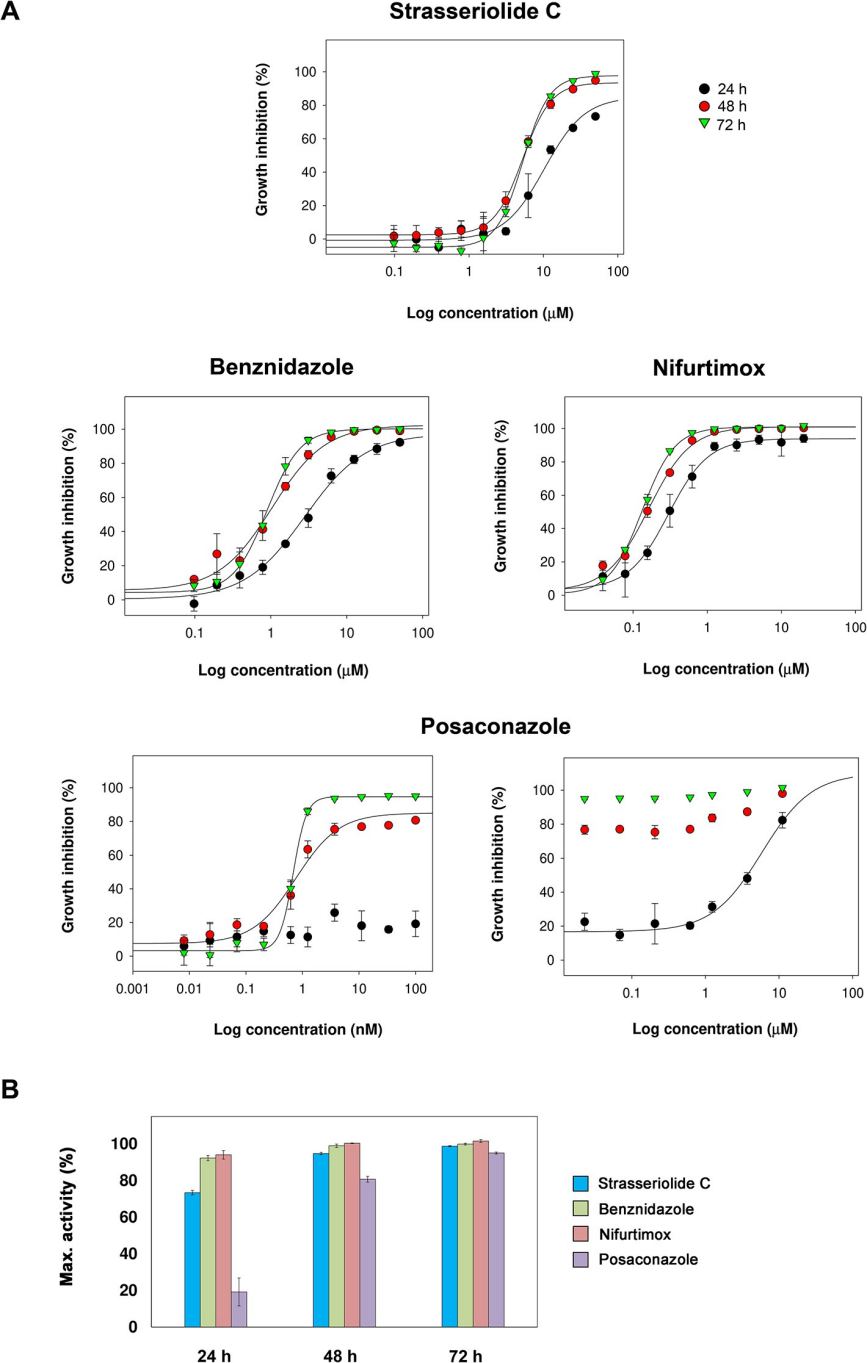
Rate of action against *T*. *cruzi* amastigotes. (A) Time-dependent correlation between compound dose and *T*. *cruzi* amastigote growth inhibition upon 24, 48 and 72 hours exposure plotted as 10-point dose-response curves. Dose-response data obtained for posaconazole after 24, 48 and 72 h exposure at higher concentrations is indicated. (B) Maximum percentage activity against intracellular forms of *T*. *cruzi* obtained after 24, 48 and 72 hours treatment with 50 μM strasseriolide C (blue bars), 50 μM benznidazole (green bars), 20 μM nifurtimox (red bars) or 100 nM posaconazole (purple bars) in rate of kill experiments with amastigote forms of the parasite.

Monitoring of amastigote growth inhibition in the rate of kill experiments using increasing doses of compound C and reference drugs was also performed (Fig 4A and Table 3). Percentages of growth inhibition obtained for intracellular forms of *T*. *cruzi* after 24, 48 and 72 hours treatment with 50 μM strasseriolide C, 50 μM benznidazole, 20 μM nifurtimox or 100 nM posaconazole are illustrated in Fig 4B.

Hence, strasseriolide C behaved as a fast-acting compound in a similar fashion to benznidazole and nifurtimox. For posaconazole, the EC_50_ value at 24 hours was significantly enhanced thus evidencing its slower action [40,43]. The high speed of action of strasseriolide C together with its pronounced activity against trypomastigote stage *T*. *cruzi* parasites are desirable properties that differentiate this compound from CYP51 inhibitors thus minimizing possibilities of relapse and increasing the probability of success in *in vivo* efficacy models [42,44,45].

### The cell cycle is not perturbed by strasseriolide treatment

Considering that strasseriolide A is moderately active and that previous *in vivo* results have revealed that strasseriolide B is toxic in a murine model [29] we centered our attention on strasseriolides C and D for morphological and cell cycle studies using *T*. *cruzi* epimastigotes. Moreover, these two novel antiprotozoal molecules have shown to be safe and fulfill a desirable pharmacokinetic profile with medium (strasseriolide C) and low (strasseriolide D) liver microsome clearance [29]. Strasseriolide C, which has medium clearance, may have enough metabolic stability for potential in vivo efficacy with lower possible liabilities of toxicity [29].

For cell cycle analysis, epimastigote replicative forms of *Trypanosoma cruzi* were exposed to two distinct inhibitory concentrations of strasseriolides C and D, EC_30_ and EC_70_, in order to analyze conditions of mild and high exposure. These values were calculated from the dose-response curves obtained with a four parameter logistic function regression fit and were 36 μM and 68 μM, respectively, in the case of compound D, and 28 μM and 47 μM for compound C. The different epimastigote *T*. *cruzi* populations observed by DAPI staining were categorized by microscopy according to the number of nuclei (N) and kinetoplasts (K). Normal cell phases such as 1N1K, 1N*1K*, 1N*2K and 2N2K together with aberrant population such as 2N1K or 0N1K were analyzed. Population distribution in untreated epimastigotes is in agreement with what was previously observed [46].

**Fig 5:**
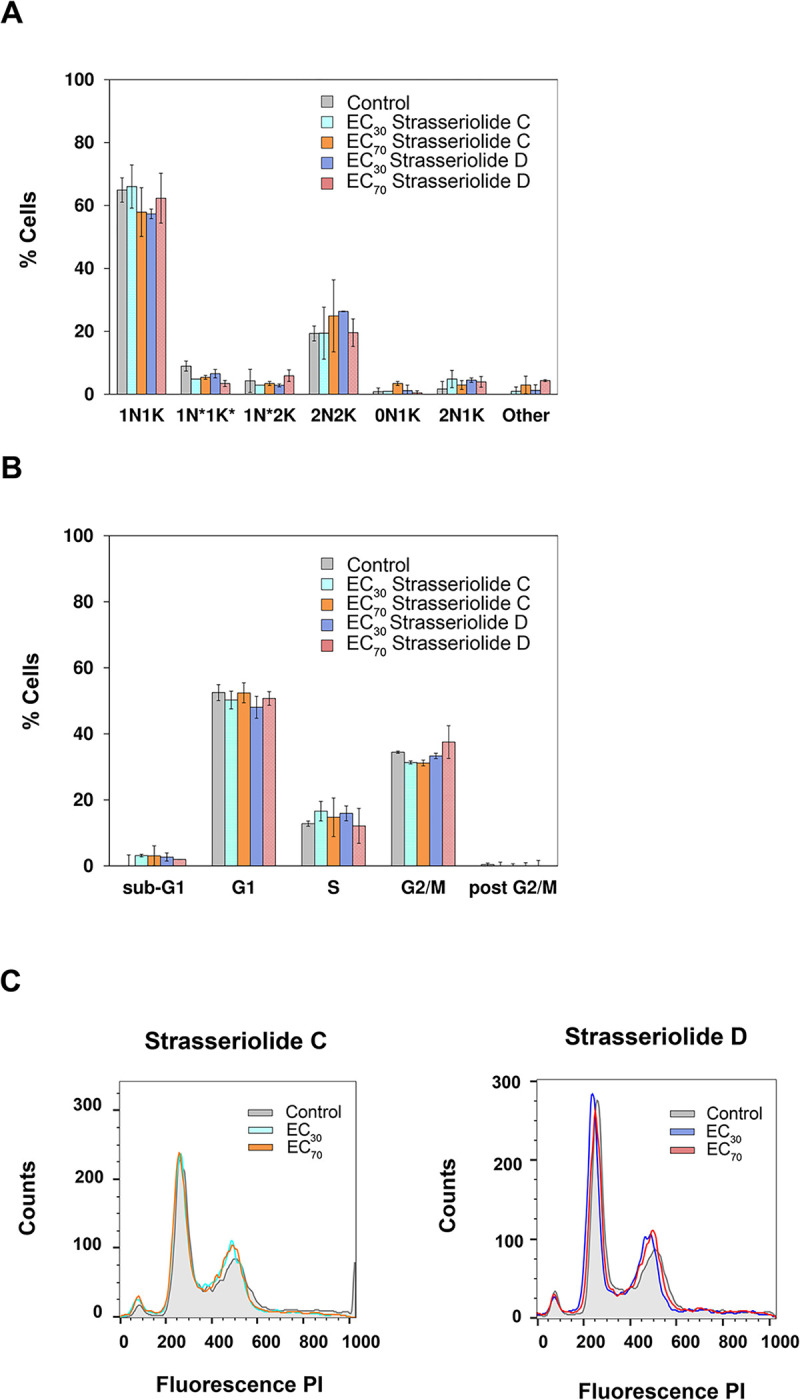
Cell cycle progression of *T*. *cruzi* epimastigotes after 72-hour drug exposure. (A) Fluorescence microscopy with DAPI staining. Bars represent the mean (± SD) calculated from two independent experiments accounting for at least 80 cells per condition respectively. (B) FACS analysis with propidium iodide staining. Bars represent the mean (± SD) calculated from two independent experiments and analyzed with Flow Jo software. (C) Overlay of fluorescence histograms obtained for control and treated fixed parasites stained with propidium iodide.

Experiments were performed with treated and non-treated parasite cultures and minimally 80 cells were analyzed for each condition. However, no significant differences in the cell cycle were identified after the comparison of treated and control parasite populations after 72 hours (Fig 5A).

A cell cycle evaluation by flow cytometry with epimastigotes treated with strasseriolides C and D rendered similar results (Fig 5B and 5C).

### Morphological studies

Size and complexity of epimastigote *T*. *cruzi* forms after 72 hours treatment were evaluated by Side (SSC) and Forward Scatter (FCS) flow cytometry. Benznidazole at EC_30_, EC_70_ and EC_90_ (7.5, 22.5 and 96 μM, respectively) concentrations was used as a control.

**Fig 6:**
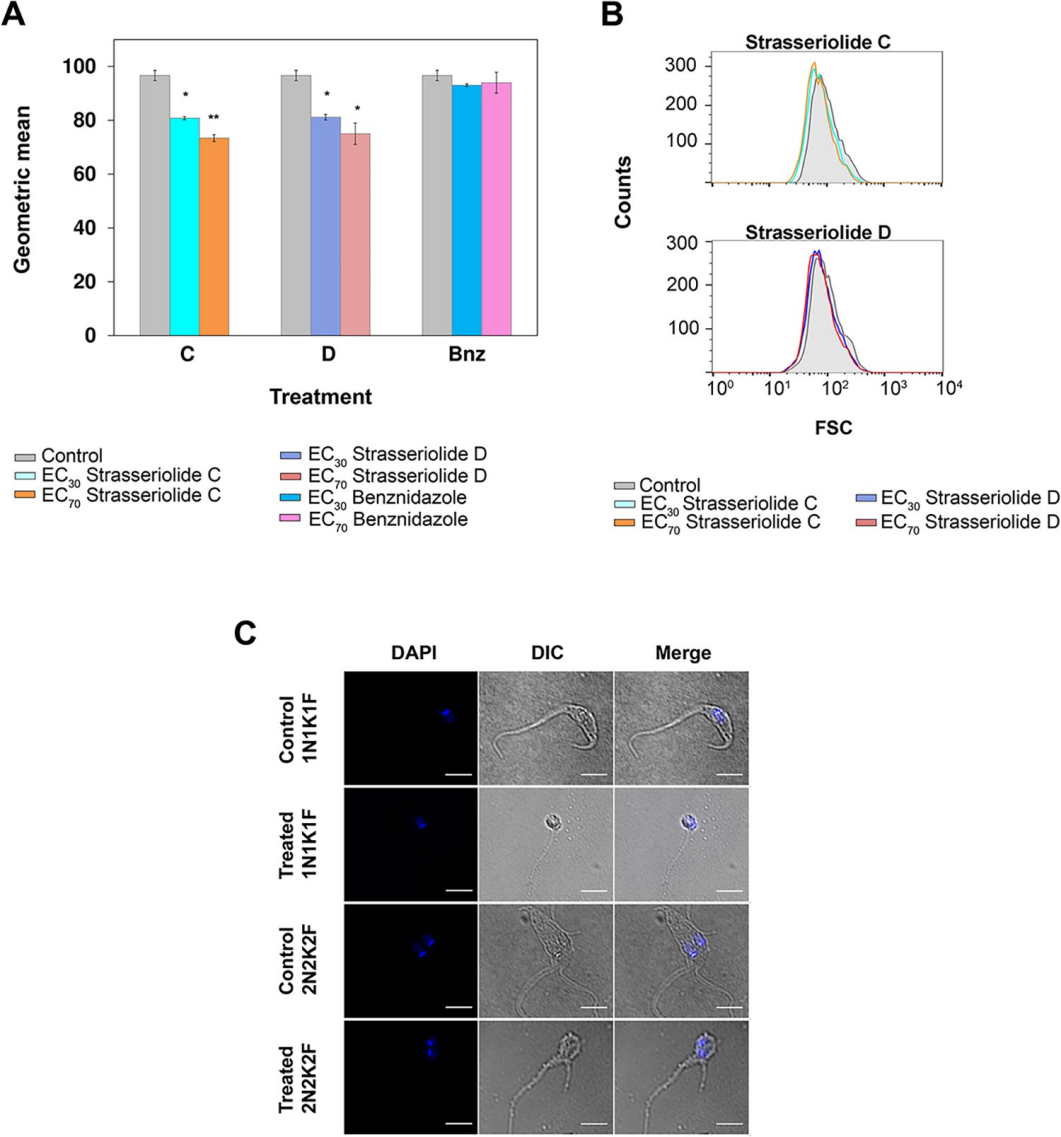
*T*. *cruzi* epimastigote size reduction after 72-hour drug exposure. (A) Cellular parameters were collected and analyzed using flow cytometry. Asterisks indicate significant differences calculated by the Student’s t-test in geometric mean values of forward scatter (FSC) intensity with respect to control parasites: * p< 0.05, ** p< 0.01. Bars represent the mean (± SD) calculated from two independent experiments and analyzed with Flow Jo software. (B) Histograms show forward scatter (FSC) intensity of cell populations treated with a single dose (EC_30_ blue or EC_70_ yellow) of strasseriolides D and C, upper and lower panel, respectively, with respect to control parasites. (C) Fluorescence and differential interference contrast (DIC) microscopy of fixed cells after 72 h treatment with strasseriolide C at EC_70_ versus control cells. Nuclei (N) and kinetoplasts (K) were stained with DAPI. Digital images were acquired with a Leica DMi8 inverted light microscope. Bars, 10 μm.

Interestingly, there is a significant decrease in the cell size of parasites treated with 30% inhibitory concentrations of strasseriolides C and D. Estimated percentage of size reduction with respect to untreated parasites was 16% in both cases. Exposure to higher concentrations (EC_70_) further enhanced perturbation of parasite size, reaching a 22% and 24% reduction after 72 hours treatment with C and D, respectively (Fig 6A and 6B), whereas benznidazole (Bnz) produced a 17.2% epimastigote size reduction but only at a lethal dose of 96 μM, similar to what has been previously described [34]. However, since there is no significant reduction at lower concentrations of benznidazole, the observations suggest specific effect of these macrolides on cell size, independent of their impact on cell proliferation.

Indeed, prominent morphological changes were visualized by differential interference contrast microscopy. Fig 6C illustrates smaller and rounded parasites after strasseriolide C exposure at EC_70_ concentrations.

The cause for the morphological defects in *T*. *cruzi* parasites observed upon exposure to these novel compounds remains to be established yet these observations could be indicative of altered membrane function or metabolic imbalance.

To determine if ATP production was affected by these compounds, we measured intracellular levels in *T*. *cruzi* epimastigotes. After 72-hour treatment with strasseriolide C at doses ranging from 0.4 to 6.25 μM, 70 μl of Cell-Titer Glo reagent were added with a multichannel pipette. Plates were incubated at 28°C for 10 minutes, after which luminescence was recorded. Luminescence relative light unit (RLU) values were interpolated in the ATP calibration curve (S4A Fig) and pmol of ATP per well were quantified. Considering proliferation inhibition due to strasseriolide C activity, values were normalized with respect to the number of parasites as determined by counting in a Neubauer chamber using phase contrast microscopy. Strasseriolide C treatment results in a discrete reduction of the cellular ATP pool (S4B Fig).

### Studies *in vivo*

Previously, strasseriolides A–D have shown no cytotoxicity, no cardiotoxicity and no drug-drug interactions *in vitro*. In addition, strasseriolides C and D have been described to exhibit *in viv*o antiprotozoal activity in a *Plasmodium berghei* murine model [29]. Taking into consideration the prominent activity of the latter compounds against *T*. *cruzi*, one of these compounds, C, was further investigated in preliminary *in vivo* efficacy experiments. Control vehicle and benznidazole were included in the study as controls for infection and effective treatment, respectively.

**Fig 7:**
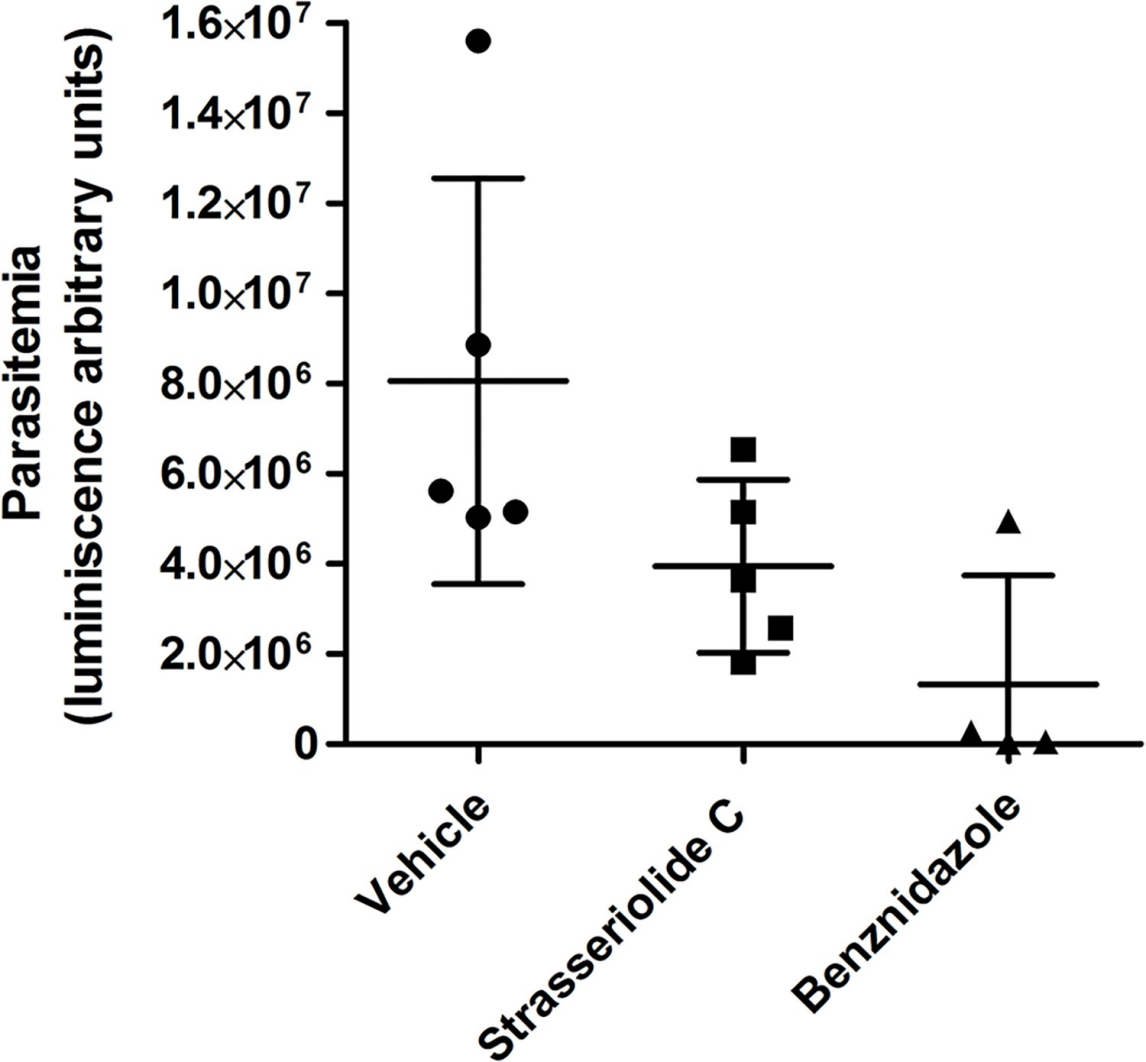
Representation of *in vivo* mice efficacy after 5 days of strasseriolide C treatment. Mice were infected with the transgenic *T*. *cruzi* line and treated at 72 h post-infection with 50 mg/kg of strasseriolide C (five replicates) for 5 consecutive days. Vehicle and 30 mg/kg benznidazole were used as controls.

A lowered parasitaemia *in vivo*, as indicated by the measurement of luminescence arbitrary units, was observed in comparison to the control vehicle (Fig 7) thus illustrating that compounds of this structural class have promising activity also in animal models of the disease.

#### Final remarks

At present, the pipeline for treatment of Chagas disease is poor and there is a pressing need for compounds with novel modes of action. In the case of new therapies for Chagas based on natural products, hundreds of plant species have been evaluated for potential antiparasitic activity while microbial natural products have been less explored [26]. Strasseriolides A–D present a unique structural scaffold that includes an 18-membered macrolide core ring structure with a carboxylate group at C1 and six methyl groups attached to C2, C6, C8, C10, C14 and C18 (Fig 1) [28]. The structural differences observed between the four compounds include the presence of oxygenated functionalities at C11 and C13 (R_1_ and R_3_ = OH) in the case of strasseriolide D, hence being the most polar, followed by a C11 hydroxy group and C13 ketone (R_1_ = OH and R_3_/R_4_ = O) in the case of strasseriolide C, hydroxylation only at C11 (R_1_ = OH) in the case of strasseriolide B, and finally a ketone function at C11 (R_1_/R_2_ = O) in the case of strasseriolide A which is the less polar molecule of the family. The seemingly slight structural differences between the four compounds translated into significant differences in their biological effects. Comparing the structures of the four compounds, the presence of a hydroxy versus a ketone group at C-11 seems to be essential for the antiparasitic activity of the molecules as strasseriolide A, having a ketone at this position, did not show significant antiprotozoal activity *in vitro*. A hydroxy group at C-11 combined with the lack of a second oxygenated functionalization at C-13 appears to confer potent antiparasitic activity on strasseriolide B, although associated with high toxicity *in vivo*. Finally, strasseriolides C and D, having oxygenated functions at both, C-11 and C-13, displayed selective *in vitro* antiprotozoal activity (against *T*. *cruzi*, *L*. *donovani* and previously against *P*. *falciparum*), ideal preclinical properties and *in vivo* parasite clearance of infected mice. It could thus be inferred from the structural differences between the four compounds that, C11-hydroxylation of this compound class is key for biological activity, but when coupled to the absence of hydroxylation/oxygenation at C13 it leads to many fold increase in bioactivity, which eventually tips the balance towards toxicity as observed for strasseriolide B.

The discovery of new drugs from natural sources entails limitations, such as the isolation in sufficient quantities and the characterization of pure compounds. However, advances in automation, novel omics and improved culture techniques are allowing for major advances. The possibility of obtaining microbial products by scaling up the fermentations of the producing microbial strain is an additional advantage. Moreover, the total synthesis of strasseriolides A and B has been recently reported [47] which provides an avenue for the design and synthesis of new strasseriolides with optimized pharmacological properties and activities.

In conclusion, studies conducted with these recently discovered potent macrolides, evidence that compounds C and D are active against both intracellular amastigote and trypomastigote forms of *T*. *cruzi in vitro*. Interestingly, compound C is fast-acting and exhibits efficacy in a mouse model of acute Chagas disease. These observations pave the way for the design and exploitation of strasseriolides as promising antiparasitic agents that may prove useful for the treatment of Chagas’ disease.

## Supporting information

S1 Data Excel spreadsheet containing, in separate sheets, all numerical values for Figs 1, 2A, 2B, 3A, 3B, 4A, 4B, 5A, 6, S2B, S3A, S3B, S4A and S4B. (XLSX)

S1 Fig HPLC-UV (210 nm) traces of pure strasseriolides A-D.
(TIF)

S2 Fig *T*. *cruzi* epimastigote viability assay and effect of DMSO. (A) Linear correlation of fluorescence versus culture density upon resazurin addition. Each point represents the mean value of two biological replicates with error bars for standard deviation. (B) Effect of DMSO. Parasites were seeded at 200,000 cells per well and grown for 72 hours. Each bar represents the mean value of nine independent cultures without DMSO (white, control), with 0.1% DMSO (grey) or 0.2% DMSO (black). Reading time was two and four hours after resazurin addition. Fluorescence reading at λexc = 550 and λem = 590 nm.
(TIF)

S3 Fig Time-dependent resazurin reduction by *T*. *cruzi* trypomastigotes at different cell densities. Trypomastigotes were obtained as indicated in the methods section and prior to the resazurin assay, were incubated for 24 (panel A) and 48 hours (panel B) at 37°C. Fluorescence was measured after 2 hours (circles) and 24 hours (triangles) of incubation with resazurin.
(TIF)

S4 Fig Quantification of intracellular ATP levels. (A) ATP standard curve used for interpolating RLU read outs. (B) T. *cruzi* epimastigotes were treated with strasseriolide C for 72 hours at increasing concentrations, and ATP content was determined using the Cell-Titer Glo luminescent viability assay and normalized by the number of cells per well. Results correspond to the mean of three replicates for each condition.
(TIF)
