# Supplementary figures and images for: Strasseriolides display in vitro and in vivo activity against trypanosomal parasites and cause morphological and size defects in Trypanosoma cruzi
Source: PLoS Negl Trop Dis. 2023 Sep 15;17(9):e0011592. doi: 10.1371/journal.pntd.0011592 (PMC10529594; doi:10.1371/journal.pntd.0011592)

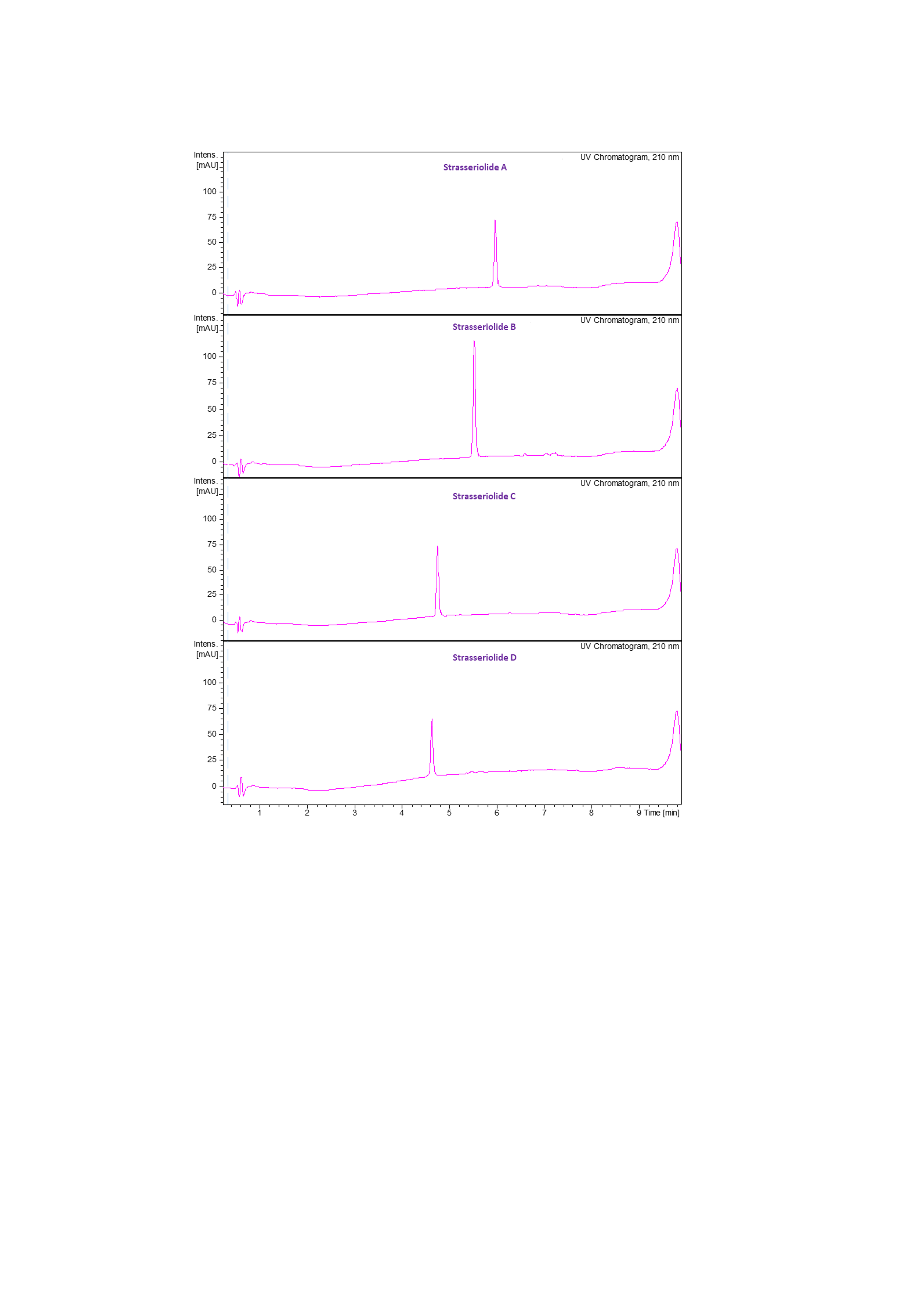

Supplement: S1 Fig — HPLC-UV (210 nm) traces of pure strasseriolides A-D. (TIF) [file pntd.0011592.s002.tif]

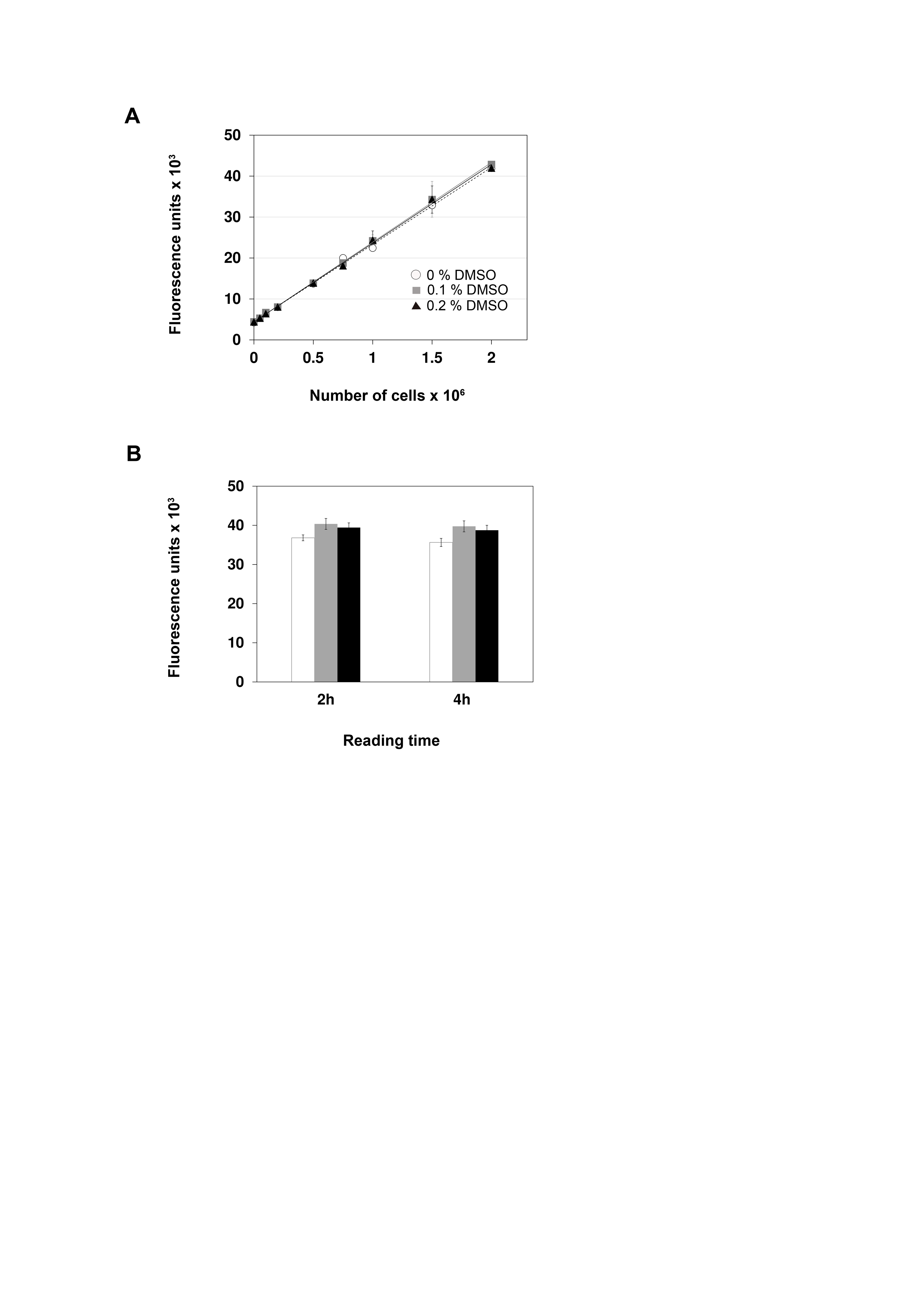

Supplement: S2 Fig — (A) Linear correlation of fluorescence versus culture density upon resazurin addition. Each point represents the mean value of two biological replicates with error bars for standard deviation. (B) Effect of DMSO. Parasites were seeded at 200,000 cells per well and grown for 72 hours. Each bar represents the mean value of nine independent cultures without DMSO (white, control), with 0.1% DMSO (grey) or 0.2% DMSO (black). Reading time was two and four hours after resazurin addition. Fluorescence reading at λexc = 550 and λem = 590 nm. (TIF) [file pntd.0011592.s003.tif]

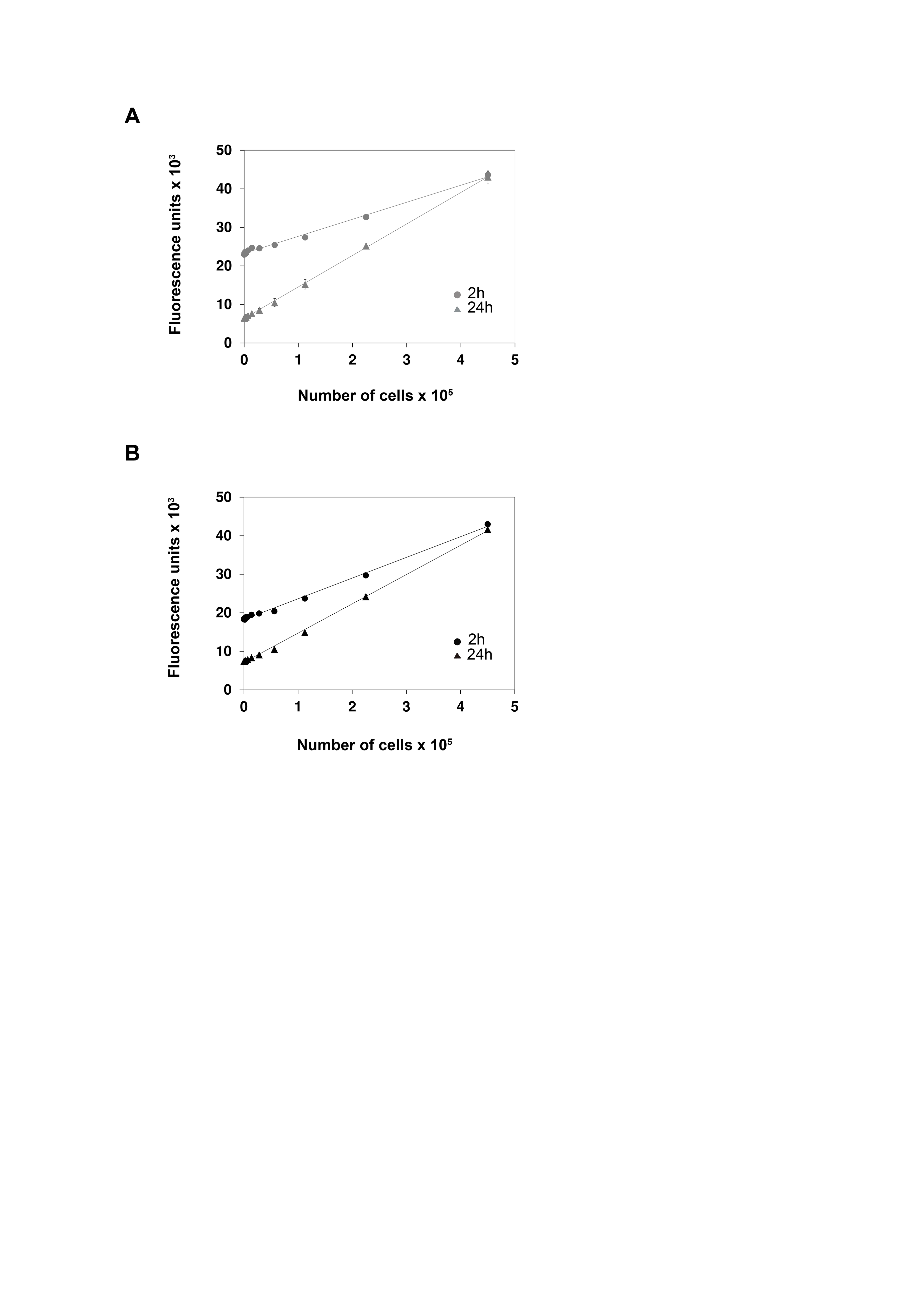

Supplement: S3 Fig — Trypomastigotes were obtained as indicated in the methods section and prior to the resazurin assay, were incubated for 24 (panel A) and 48 hours (panel B) at 37°C. Fluorescence was measured after 2 hours (circles) and 24 hours (triangles) of incubation with resazurin. (TIF) [file pntd.0011592.s004.tif]

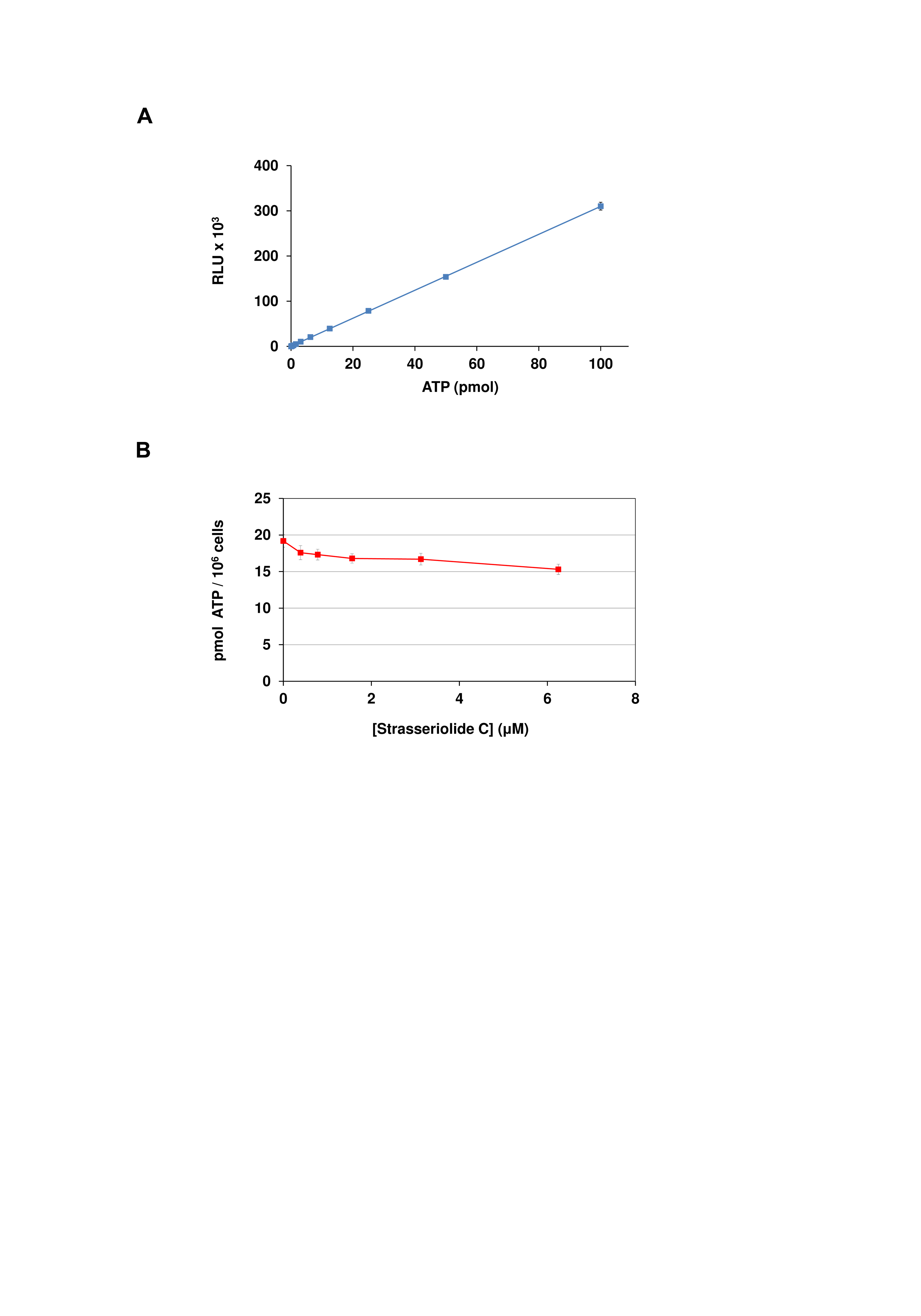

Supplement: S4 Fig — (A) ATP standard curve used for interpolating RLU read outs. (B) T. cruzi epimastigotes were treated with strasseriolide C for 72 hours at increasing concentrations, and ATP content was determined using the Cell-Titer Glo luminescent viability assay and normalized by the number of cells per well. Results correspond to the mean of three replicates for each condition. (TIF) [file pntd.0011592.s005.tif]
